# 

**Published:** 1844-10

**Authors:** 


### CONTENTS

### OF NO. X.

### ORIGINAL COMMUNICATIONS.

### ESSAYS AND CASES.

Art. I.—Experimental Researches concerning Febrile Caloricity, both before and after death—Post-mortem Fever. By Bennet Dowler, M.D., of New Orleans. - - - - - - - - - 281
Art. II.—A Case of White Swelling cured by Iodine. By Dr. J. P. Anderson, of Sparta Alabama. - - - - - - - - - 299

### REVIEWS.

Art. III.—Lectures on the Theory and Practice of Midwifery, delivered in the Theatre of St. George's Hospital. By Robert Lee, M. D., F.R.S., &c. Illustrated with numerous wood engravings.
A Practical Treatise on Midwifery; exhibiting the present advanced state of the Science. By F. J. Moreau, Officer of the Legion of Honor, and of the Order of Leopold; Professor of Midwifery and of the Diseases of Women and Children in the Faculty of Medicine of Paris, &c. Translated from the French by Thomas Forrest Betton, M. D., and edited by Paul B. Goddard, A.M., M.D., &c. With 80 plates, comprising numerous separate illustrations. - - - - - - - - - 302
Art. IV.—On the Superstitions connected with the History and Practice of Medicine and Surgery. By Thomas Joseph Pettigrew, F. R.S., F.S.A., Doctor of Philosophy of the University of Gottingen, Surgeon to H. R. H. the Duchess of Kent, to the Asylum for Female Orphans, &c., &c., &c. - - - - - - - 317
Art. V.—A System of Human Anatomy, General and Special. By Erasmus Wilson, M.D., Lecturer on Anatomy, London. Second American edition, by Paul B. Goddard, A.M., M.D., &c. With over 200 illustrations by Gilbert. From the second London edition. - - - - - - - - - - - - - - - 325
Art. VI.—The American Journal of Insanity: edited by the Officers of the New York State Lunatic Asylum, - - - - - - 326

ART. VII.—Human Health; or the Influence of Atmosphere and Locality; Change of Air and Climate; Seasons; Food; Clothing; Bathing and Mineral Springs; Exercise; Sleep; Corporeal and Intellectual Pursuits, &c., on healthy Man; constituting Elements of Hygiene. By ROBLEY DUNGLISON, M.D., Professor of the Institutes of Medicine, &c., in Jefferson Medical College of Philadelphia, &c., &c. A new edition, with Modifications and Additions. 330
ART. VIII.—The Principles and Practice of Modern Surgery. By ROBERT DRUITT, Surgeon. From the third London edition. Illustrated with 153 wood engravings. With Notes and Comments: by JOSHUA B. FLINT, M.D., M.M., S.S., late Professor of Surgery in the Medical Institute of Louisville. - - - - - - 332
ART. IX.—The Cyclopædia of Practical Medicine. Edited by JOHN FORBES, M. D., F. R. S., ALEX. TWEEDIE, M. D., F. R. S., and JOHN CONOLLY, M.D. Revised with additions, by ROBLEY DUNGLISON, M.D. Parts VIII., IX., X., and XI. - - - - - - 335
ART. X.—A Treatise on Hare-Lip, and its Treatment. By Dr. S. P. HULLIHEN, of Wheeling, Virginia. - - - - - - - 335
ART. XI.—A Manual of Examinations, upon Anatomy and Physiology, Surgery, Practice of Medicine, Chemistry, Materia Medica, Obstetrics, &c. Designed for the use of Students of Medicine throughout the United States. By J. L. LUDLOW, M D. - - 339
ART. XII.—Anatomical Atlas; illustrative of the Structure of the Human Body. By HENRY H. SMITH, M.D., &c.; under the Supervision of WILLIAM E. HORNER, M.D , &c. - - - - - - - - 339
ART. XIII.—An Attempt to Explain the Nature of Electricity and its Intention in the Economy of the Universe. By ROBERT SERRELL WOOD. - - - - - - - - - - - - - - - 340

### SELECTIONS FROM AMERICAN AND FOREIGN JOURNALS.

The Valerianate of Zinc. - - - - - - - - - - - 341
Antagonism of Pulmonary Phthisis and of Intermittent Fever. - - 341
Rice-grounds. - - - - - - - - - - - - - . - - 342
Hydrophobia treated by the poison of the Viper - - - - - - 343
On the mortality at different ages in certain diseases. - - - - 343
Dr. Boling on Quinine in Fevers and Inflammation. - - - - 348
Longet on Phrenology. - - - - - - - - - - - - - 350
Partus Siccus. - - - - - - - - - - - - - - 351
Professional Justice to the Profession. - - - - - - - - 351
Anomalies of the Teeth. - - - - - - - - - - - 353

### ORIGINAL INTELLIGENCE.

Traveling Letter, No. V, - - - - - - - - - - - 354
Traveling Letter, No- VI. - - - - - - - - - - - 360
Dr. M'Dowell and his pretensions to the Cure of Consumption. . - 366
Medical Examiner and Western Medical Schools. - - - - - 369
A New Medicinal Vegetable. - - - - - - - - - - 370
The Health of the Clergy. - - - - - - - - - - - 371
Louisville Summer School of Medicine - - - - - - - . - 372
Medical Institute of Louisville. - - - - - - - - - 372
To the Subscribers of this Journal. - - - - - - - - 372

